# Cardioprotective properties of leptin in patients with excessive body mass

**DOI:** 10.1007/s11845-020-02211-9

**Published:** 2020-03-20

**Authors:** Aleksandra Paduszyńska, Agata Sakowicz, Maciej Banach, Marek Maciejewski, Marek Dąbrowa, Agata Bielecka-Dąbrowa

**Affiliations:** 1 Department of Hypertension, Chair of Nephrology and Hypertension, Medical University of Lodz, Lodz, Poland; 2 Department of Medical Biotechnology, Medical University of Lodz, Lodz, Poland; 3 Department of Cardiology and Congenital Diseases of Adults, Polish Mother’s Memorial Hospital Research Institute (PMMHRI), Lodz, Poland; 4 Department of Biopharmacy, Chair of Biopharmacy, Medical University of Lodz, Lodz, Poland

**Keywords:** Adipokines, Hypertrophy, Left ventricular remodeling, Leptin

## Abstract

**Background:**

Adipose tissue is producing adipokines that play different roles in the pathophysiology of cardiovascular disease.

**Aims:**

The study aimed to assess the role of selected biomarkers in hypertensive patients with overweight and obesity compared with those with normal body-mass index (BMI).

**Methods:**

A total of 62 patients with BMI < 25 kg/m^2^ (median age 54 (46–58) yrs., 57% males) and 51 with BMI ≥ 25 kg/m^2^ (median age 53 (48–59) yrs., 37% males) were enrolled. Biochemical parameters, leptin, adiponectin, and resistin; asymmetric dimethylarginine; interleukin 6; and N-terminal propeptide of type III procollagen, were assessed in plasma. The evaluation of hemodynamic parameters was performed using SphygmoCor 9.0 tonometer. Echocardiography was performed using AlokaAlpha 10 Premier device.

**Results:**

Overweight and obese patients had significantly higher concentration of leptin (34 vs 18 ng/ml; *p* = 0.03), ADMA (0.43 vs 0.38 μmol/l, *p* = 0.04), and lower concentration of adiponectin (5.3 vs 7 μg/ml, *p* = 0.01). The only significant difference in tonometry analysis was higher aortic pulse pressure (mmHg) in patients with BMI ≥ 25 kg/m^2^ group (34 vs 30; *p* = 0.03). These patients had also significantly lower peak systolic velocity and early diastolic velocity in tissue Doppler imaging of the right ventricle free wall at the level of the tricuspid annulus compared with controls (*p* = 0.02 and *p* = 0.001, respectively). The level of leptin is correlated negatively with the left ventricular mass index (LVMI) (*R* Spearman = − 0.5; *p* = 0.002) and PWV (*R* = − 0.4; *p* = 0.01) and ADMA with total and LDL cholesterol (*R* = − 0.42; *p* = 0.008), and adiponectin is correlated positively with HDL cholesterol (*R* = 0.67; *p* = 0.0001).

**Conclusions:**

Leptin concentrations were inversely proportional to LVMI and PWV in patients with BMI < 25 kg/m^2^.

**Trial registration:**

Clinicaltrials.gov study ID: NCT04175080.

## Introduction

Excessive body mass is the increasing health problem all over the world. The analysis of data collected between 1990 and 2015 revealed that cardiovascular disease was the major cause of deaths related to high body-mass index (BMI) [1]. It is the result of different changes caused by the excessive fat accumulation. Besides being an energy storage, the adipose tissue is also an endocrine organ producing adipokines like leptin, resistin, adiponectin, and interleukin 6 (IL-6) [2]. Among them particular attention is paid to leptin. This study aimed to assess the clinical role of selected biomarkers in patients with overweight and obesity compared with those with normal body-mass index (BMI).

## Material and methods

A total of 113 hypertensive pharmacologically treated patients were enrolled to this one center study. Based on the BMI, they were divided into two groups: with BMI < 25 kg/m^2^ and with BMI ≥ 25 kg/m^2^. Basic characteristic of studied groups of patients is presented in Table 1.

**Table 1:** Basic characteristics of studied groups of patients

Parameter	Patients with BMI < 25 kg/m^2^	Patients with BMI ≥ 25 kg/m^2^	*p*
Number of patients	62	51	
Median age (years)	54 (46–58)	53 (48–59)	0.46
Gender (male)	57%	38%	0.051
BMI (kg/m^2^)	22.8 (20.8–24.5)	28.7 (27.2–32.9)	< 0.001
BSA (m^2^)	1.76 (1.65–1.95)	1.98 (1.87–2.12)	< 0.001
Carbohydrate disturbances	53%	67%	0.147

*BMI*, body-mass index; *BSA*, body surface area

Data regarding pharmacotherapy used in the patients groups are summarized in Table 2.

**Table 2:** Pharmacotherapy in the studied groups of patients

Drug class	Patients with BMI < 25 kg/m^2^	Patients with BMI ≥ 25 kg/m^2^	*p*
Beta-blockers	10 (18.52%)	12 (27.27%)	0.423
ACE inhibitors/ARBs	14 (25.93%)	18 (40.91%)	0.116
Calcium channel blockers	7 (12.96%)	9 (20.45%)	0.470
Diuretics	7 (12.96%)	11 (25.00%)	0.205
Statins	7 (12.73%)	14 (31.11%)	0.046
ASA	8 (14.55%)	10 (22.22%)	0.464
Insulin	1 (1.82%)	4 (9.09%)	0.168

*ACE*, angiotensin-converting enzyme; *ARB*, angiotensin receptor blocker; *ASA*, acetylsalicylic acid

In all patients, the following biochemical parameters were measured:

- Selected adipokines: Leptin, adiponectin, resistin, and IL-6
- An endogenous inhibitor of nitric oxide synthase: Asymmetric dimethylarginine (ADMA)
- Biomarker of cardiac extra-cellular matrix turnover: N-terminal propeptide of type III procollagen(PIIINP)
- Lipids
- Glucose

They underwent also non-invasive assessment of hemodynamic parameters as well as echocardiography.

### Biochemical parameters

Blood samples for laboratory tests were collected from patients assigned to either group in a hospital setting, thus minimizing the risk of infection in both the subject and the person collecting the sample. Laboratory tests were performed in fasting subjects in laboratory of WAM Hospital, following a minimum 12-h period after the last meal. At the initial time point of the study, 19.5 mL of blood were collected with a vacuum blood collection system from the basilic vein for routine laboratory tests. The serum samples were centrifuged and pipetted into Eppendorf tubes and subsequently placed in a freezer (first at − 25 °C and then at − 70 °C). After the desired number of samples were obtained and their prior thawing (immediately before testing), the biochemical parameters were assessed.

#### Adipokines, ADMA, and PIIINP

The Enzyme Linked-Immunosorbent Assay (ELISA) tests were conducted for quantitative determination of resistin (BioVendor, Czech Republic), adiponectin (BioVendor, Czech Republic), leptin (BioVendor, Czech Republic), IL-6 (Gen-Probe, France), ADMA (Immunodiagnostic, Bensheim, Germany), and PIIINP (Cloud-Clone Corp, China) in human serum.

#### Lipids

Triglycerides, total, and HDL-C levels were evaluated using the AU680 device (Beckman Coulter Poland, Warsaw, Poland). The TC was measured enzymatically with standardized calibrators: cholesterol esterase and oxidase, respectively, according to the manufacturer’s specifications. The HDL-C was measured enzymatically with lipoproteins immune complex with standardized calibrators: cholesterol esterase and oxidase. LDL-C concentrations were calculated by Friedewald’s formula: LDL-C (mmol/L) = TC − HDL-C − TG/2.2. TG was measured enzymatically with glycerol phosphate oxidase and H202 determination in the presence of peroxidase.

#### Glucose

Plasma glucose was measured by ultraviolet photometry using the enzymatic reactions catalyzed by hexokinase, using Olympus reagent kits (Cat. No. OSR61221, OSR6221).

### Hemodynamic parameters

Non-invasive evaluation was performed using SphygmoCor 9.0 tonometer (AtCor Medical, Sydney, Australia). The aortic systolic pressure (SP aortic), aortic diastolic pressure (DP aortic), and aortic pulse pressure (PP aortic) were obtained. We assessed also the parameters of arterial stiffness: augmentation pressure (AP), augmentation index (AIx), and pulse wave velocity (PWV). AP was calculated as the difference between the first and second systolic peaks on the central pressure waveform, which was derived by radial applanation tonometry and application of a generalized transfer function to the radial pressure waveform. AIx was calculated by AP as a percentage of the total pressure waveform height. PWV was assessed using electrocardiogram-gated sequential tonometry at the carotid and femoral sites and calculated as the path length divided by transit time (meter/s). Path length is a result of the subtraction of the distance between sternal notch and carotid recording site from the distance between sternal notch and femoral site.

### Echocardiography

Echocardiography was performed using Alpha 10 Premier device (ALOKA, Tokyo, Japan) with a 3–11 MHz probe. Quantitative assessment was used according to current guidelines. The ratio of peak velocity of early diastolic transmitral flow to peak velocity of early diastolic mitral annular motion as determined by pulsed-wave Doppler (E/E′) was calculated as an index of the left ventricular filling pressure. The left ventricular ejection fraction (LVEF) was determined by biplane Simpson’s method. The left atrial volume index (LAVI) was calculated by dividing left atrial volume by body surface area. The peak systolic velocity (Sm) and early diastolic velocity (e′) were assessed using tissue Doppler.

### Statistical analysis

The STATISTICA 10 software package (StatSoft, Poland), SPSS 21, R-project 3.0.1 (packages: rmsipredictAbel), was used for analysis. Results were considered significant if *p* < 0.05. The Shapiro-Wilk test was used to assess the normality of distribution. To compare two groups, Student’s *t* test for continuous variables with normal distribution and Mann-Whitney *U* test for non-normally distributed variables were used. For quantitative variables (continuous and discrete) to evaluate correlations between variables, Spearman’s rank correlation coefficient was used.

## Results

### Biochemical parameters

In comparison with patients with proper body weight, those with BMI ≥ 25 had significantly higher concentrations of total leptin, ADMA, and glucose. There were no statistically significant differences in resistin, IL-6, PIIINP, and total cholesterol concentrations between groups. Overweight and obese patients had significantly lower concentrations of adiponectin, LDL, and HDL cholesterol. The results of measurements are presented in Table 3.

**Table 3:** Evaluation of biochemical parameters among the groups

Parameter	Patients with BMI < 25 kg/m^2^	Patients with BMI ≥ 25 kg/m^2^	*p*
Adipokines:			
- Leptin (ng/ml)	18.66 (5.95–51.75)	34.53 (16.93–70.49)	**0.03**
- Adiponectin (μg/ml)	7.07 (5.03–11.12)	5.36 (3.90–8.62)	**0.02**
- Resistin (ng/ml)	4.53 (3.04–6.25)	3.95 (3.14–6.92)	0.96
ADMA (μmol/l)	0.38 (0.34−0.46)	0.43 (0.37 − 0.53)	**0.04**
IL-6 (pg/ml)	2.53 (0.76–6.01)	1.42 (0.56–3.38)	0.06
PIIINP (ng/ml)	15.21 (13.45–17.73)	15.59 (13.54–21.07)	0.62
Glucose (mmol/L)	5.57 (5.10–6.20)	6.00 (5.41–6.70)	**0.01**
LDL cholesterol (mmol/l)	3.01 (2.64–3.67)	2.80 (1.95–3.83)	**0.02**
HDL cholesterol (mmol/l)	1.33 (1.13–1.60)	1.19 (0.94–1.44)	**0.01**
Total cholesterol (mmol/l)	4.98 ± 1.34	4.48 ± 1.22	0.17

For the parameters with non-normal distribution, there are given median values (lower and higher values). For the parameter with normal distribution there are given mean values ± standard deviation (SD). *ADMA*, asymmetric dimethylarginine; *IL-6*, interleukin 6; *PIIINP*, N-terminal propeptide of type III procollagen; *LDL*, low density lipoprotein; *HDL*, high density lipoprotein

### Hemodynamic parameters

The only significant difference (*p* = 0.03) in tonometry analysis was lower APP in BMI **≥** 25 group in comparison with the control group. The differences between groups in aortic pressures and the parameters of arterial stiffness, AP, AIx, AIx@HR75, and PWV, were insignificant. The values of assessed parameters are compared in Table 4.

**Table 4:** Evaluation of hemodynamic and echocardiographic parameters among the groups

Parameter	Patients with BMI < 25 kg/m^2^	Patients with BMI ≥ 25 kg/m^2^	*p*
DP aortic (mmHg)	81 (75–88)	85 (78–92)	0.37
SP aortic (mmHg)	119.94 ± 18.68	124.47 ± 17.41	0.37
AP (mmHg)	8 (5–17)	11 (7–16)	0.97
AIx (%)	24.67 ± 13.63	23.65 ± 11.07	0.78
AIx@HR75	21.48 ± 11.95	20.88 ± 11.09	0.85
PWV	6.9 (6.0–8.4)	7.6 (6.3-8.9)	0.29
APP (mmHg)	34.0 (29.5–38.5)	30.0 (23.0–38.0)	**0.03**
E/E′	6.68 (5.53–8.00)	7.30 (6.00–8.84)	0.18
LAVI (ml/m^2^)	24.92 ± 10.52	23.25 ± 8.04	0.57
LVEF (%)	60.0 (57.5–65.0)	60.0 (55.0–64.0)	0.23
LVMI (g/m^2^)	101 (83–119)	104 (87–117)	0.88
Sm (cm/s)	13.80 (12.00–16.70)	12.65 (11.00–15.00)	**0.02**
e′ (cm/s)	14.66 ± 4.38	12.13 ± 2.15	**0.003**

For the parameters with non-normal distribution, there are given median values (lower and higher values). For the parameters with normal distribution, there are given mean values ± standard deviation (SD). *DP*, aortic, diastolic pressure aortic; *SP*, aortic, systolic pressure aortic; *AP*, augmentation pressure; *AIx*, augmentation index; *AIx@HR75*, adjusted augmentation index at heart rate 75 per minute; *PWV*, pulse wave velocity; *APP*, aortic pulse pressure; *E/E′*, ratio of peak velocity of early diastolic transmitral flow to peak velocity of early diastolic mitral annular motion as determined by pulsed wave Doppler; *LAVI*, left atrial volume index; *LVEF*, left ventricular ejection fraction; *LVMI*, left ventricular mass index; *Sm*, peak systolic velocity; *e′*, early diastolic velocity

### Echocardiography

As shown in Table 3, overweight and obese patients had significantly lower peak systolic velocity (*p* = 0,026) and early diastolic velocity (*p* = 0,002/*p* = 0,003, depending on the used statistical model) in tissue Doppler imaging of RV free wall at the level of the tricuspid annulus compared with the controls. There were no differences in E/E′, LAVI, LVEF, and LVMI between groups. The results of evaluated parameters are presented in Table 4.

### Correlations

**Fig. 1:**
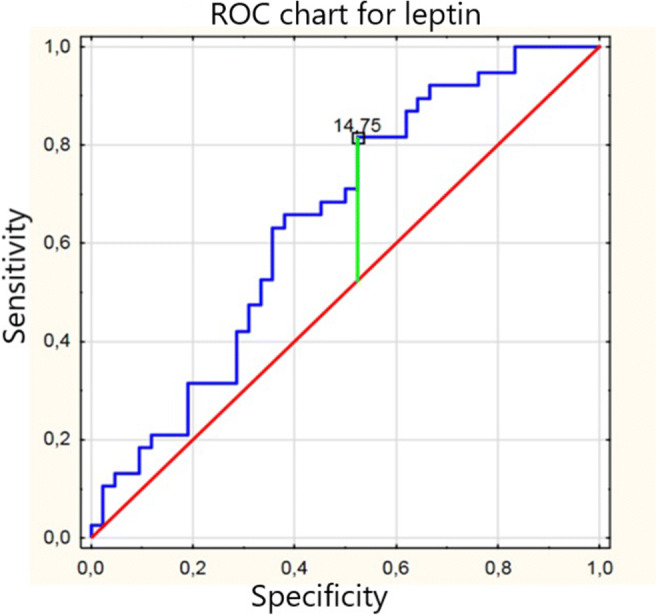
ROC chart for leptin

We determined the correlation between leptin, adiponectin, and ADMA with selected parameters in patients with BMI **≥** 25. According to results shown in Table 5, we found negative correlations of leptin with LVMI and PWV, as well as ADMA with total cholesterol and positive correlation of adiponectin with HDL cholesterol. The Roc chart for leptin is presented on Fig. 1.

**Table 5:** Spearman’s rank correlation of leptin, adiponectin and ADMA with selected parameters in patients with BMI **≥** 25

	Leptin	Adiponectin	ADMA
LDL cholesterol	ns	ns	− 0.38 (*p* = 0.02)
HDL cholesterol	ns	0.67 (*p* = 0.0001)	ns
Total cholesterol	ns	ns	− 0.42 (*p* = 0.008)
PWV	− 0.4 (*p* = 0.01)	ns	ns
LVMI	− 0.5 (*p* = 0.002)	ns	ns

*LDL*, low density lipoprotein; *HDL*, high density lipoprotein; *PWV*, pulse wave velocity; *LVMI*, left ventricular mass index; ns, not significant

## Discussion

Our study focuses on the clinical role of selected biomarkers in hypertensive patients with overweight and obesity compared with those with normal BMI. In both groups, the concentration of PIIINP suggests the risk of heart failure [3, 4]. We found a positive correlation between adiponectin and HDL and a negative correlation between ADMA and LDL and total cholesterol. We also observed potential influence of leptin on cardiac remodeling in hypertensive patients.

The observed significant difference in adiponectin concentrations between the studied groups corresponds to the lower concentrations of HDL in the obese and overweight patients. Our findings are consistent with the published data obtained from the studies on larger population. Similar results were also noted in the study in patients with type 2 diabetes [5]. However the analysis performed by Borges MC et al. [6] indicates the limited clinical benefits of this correlation. The lack of potential therapeutic use resulted from the unestablished direct effect of adiponectin on lipid profile. Probably adiponectin concentrations are reflecting other factors causing the changes in lipid profile or are dependent on a disease.

The groups in our study differ also significantly in ADMA concentrations, and in obese and overweight patients, we found a negative correlation between ADMA and LDL and total cholesterol. Surprisingly the concentrations of LDL and total cholesterol were lower in the patients whose BMI exceeds 25 kg/m^2^ (possible reason is the effect of statins used more frequently in this group). These results are in contrary to the established role of ADMA as a cardiovascular risk marker [7, 8]. The study on larger population including patients before and during dyslipidemia therapy is needed to formulate unequivocal conclusion.

Obtained results indicate protective role of leptin in LV remodeling.

Leptin has complex and pleiotropic effects, which are not fully explained [9, 10]. The recent reviews aim to summarize current knowledge and present network of signaling pathways as well as interactions with cardiovascular system [11–13]. Translation of the research results into the clinical practice is an opportunity for better prevention and treatment. However, it has not been resolved which of the leptin effects outweigh, adverse whether cardioprotective. Available non-clinical data are conflicting. They indicate either the role of leptin in the cardiomyocytes hypertrophy [14, 15] or its antihypertrophic effect [16, 17]. These differences are probably related to studies’ design. Also the results of clinical observations are equivocal and depend on many factors, for example, the characteristic of studied groups. The elevated concentration of leptin is confirmed in patients with heart failure, both with reduced and preserved ejection fraction [18]. Hyperleptinemia occurs also in majority of overweight and obese patients, but it is accompanied by resistance to leptin action [19].

Our observations are in line with large epidemiological study in which high levels of leptin were not associated with higher incidence of cardiovascular disease [20], whereas some studies on smaller groups indicate pro-hypertrophic effect of leptin [21–23]. Although it was found that hypertrophic effect of leptin is independent from the blood pressure values [21, 22], high blood pressure has the important role in developing LV hypertrophy [24]. In our study, diastolic and systolic pressure values do not differ significantly among the groups, and either patients with BMI < 25 kg/m^2^ or patients with BMI ≥ 25 kg/m^2^ had properly pharmacologically controlled hypertension. It is suggested that in the overweight and obese patients, the influence of leptin on the satiety is blocked, whereas resistance on other effects does not occur. Study of larger group is needed to assess whether the normal blood pressure in overweight and obese patients is an effect of therapy or a resistance to leptin pressor action. Data reviewed by Mark AL [25] indicate variation in leptin gene and receptor as possible causes of differences in blood pressure response. Moreover the administration of leptin does not result in the increase of blood pressure.

Another factor that can contribute to the obtained results is the localization of leptin synthesis. Plasma concentration of leptin corresponds mainly to its synthesis by adipocytes. Recent study on murine model suggests that the hypertrophic action is related to the overexpression of cardiac leptin [26]. The limitations of available measurement methods make it impossible to determine the concentration of cardiac leptin in samples from patients. However, it cannot be excluded that the observed lack of LV remodeling is the effect of low cardiac leptin synthesis in the studied obese and overweight patients. The relationship between leptin, abdominal adiposity, and arterial stiffness was assessed on large group of patients by Windham et al. [27]. The results suggest that increased pulse wave velocity associated with leptin do not depend on abdominal obesity. Beside the production by body adipose tissue, the increased concentrations of leptin may be also a result of other factors, for example, genetic.

The limitations of our study such as small sample size, characteristic of studied groups (including sex differences), and study methods should be taken into consideration while formulating the conclusions. However, the protective role of leptin in overweight and obese patients was also found in the study of Kamimura et al. [28]. The significant effect on LV hypertrophy and stiffness was reported in black obese women. Left ventricular mass was lower (*p* = 0,008 in model adjusted for age and BMI and *p* = 0,004 in model adjusted for age, BMI, diabetes, coronary heart disease, systolic blood pressure, antihypertensive medication, glomerular filtration rate, and smoking status) and diastolic wall strain was higher (*p* = 0,004 and 0,007, respectively).The same influence of leptin was observed in men but was not statistically significant [28]. The results of the study of Lieb et al. [29] also support our observations. Echocardiographic characteristic and leptin concentration of 432 older patients (> 70 years of age) with the mean BMI > 25 kg/m^2^ were evaluated. The higher concentration of leptin was associated with lower left ventricular mass and wall thickness as well as left atrial size [29].

## Conclusions

Higher leptin concentrations were inversely proportional to left ventricular mass index and pulse wave velocity in patients with overweight and obesity. Further studies are necessary to confirm these results and to establish the potential cardioprotective factors in patients whose BMI ≥ 25 kg/m^2^.
